# SARS-CoV2 pneumonia patients admitted to the ICU: Analysis according to clinical and biological parameters and the extent of lung parenchymal lesions on chest CT scan, a monocentric observational study

**DOI:** 10.1371/journal.pone.0308014

**Published:** 2024-09-19

**Authors:** Abed al Hadi Krisht, Kévin Grapin, Romain Chauvot de Beauchene, Benjamin Bonnet, Lucie Cassagnes, Bertrand Evrard, Mireille Adda, Bertrand Souweine, Claire Dupuis

**Affiliations:** 1 CHU Clermont-Ferrand, Service de Médecine Intensive et Réanimation, Clermont-Ferrand, France; 2 CHU Clermont-Ferrand, Service de Radiologie, Clermont-Ferrand, France; 3 CHU Clermont-Ferrand, Service d’Immunologie, Clermont-Ferrand, France; 4 Université Clermont Auvergne, Laboratoire d’Immunologie, ECREIN, UMR1019 UNH, UFR Médecine de Clermont-Ferrand, Clermont-Ferrand, France; 5 Université Clermont Auvergne, Unité de Nutrition Humaine, INRAe, CRNH Auvergne, Clermont Ferrand, France; 6 Université Clermont Auvergne, CNRS, LMGE, Clermont-Ferrand, France; Kaohsuing Medical University Hospital, TAIWAN

## Abstract

**Background:**

CT-scan and inflammatory and coagulation biomarkers could help in prognostication of COVID-19 in patients on ICU admission.

**Objective:**

The objectives of this study were to measure the prognostic value of the extent of lung parenchymal lesions on computed tomography (CT) and of several coagulation and inflammatory biomarkers, and to explore the characteristics of the patients depending on the extent of lung parenchymal lesions.

**Design:**

Retrospective monocentric observational study achieved on a dataset collected prospectively.

**Setting:**

Medical ICU of the university hospital of Clermont-Ferrand, France.

**Patients:**

All consecutive adult patients aged ≥18 years admitted between 20 March, 2020 and 31 August, 2021 for COVID-19 pneumonia.

**Interventions:**

Characteristics at baseline and during ICU stay, and outcomes at day 60 were recorded. The extent of lung parenchyma lesions observed on the chest CT performed on admission was established by artificial intelligence software.

**Measurements:**

Several clinical characteristics and laboratory features were collected on admission including plasma interleukin-6, HLA-DR monocytic–expression rate (mHLA-DR), and the extent of lung parenchymal lesions. Factors associated with day-60 mortality were investigated by uni- and multivariate survival analyses.

**Results:**

270 patients were included. Inflammation biomarkers including the levels of neutrophils, CRP, ferritin and Il10 were the indices the most associated with the severity of the extent of the lung lesions. Patients with more extensive lung parenchymal lesions (≥ 75%) on admission had higher CRP serum levels. The extent of lung parenchymal lesions was associated with a decrease in the PaO2/FiO2 ratio(p<0.01), fewer ventilatory-free days (p = 0.03), and a higher death rate at day 60(p = 0.01). Extent of the lesion of more than 75% was independently associated with day-60 mortality (aHR = 1.72[1.06; 2.78], p = 0.03). The prediction of death at day 60 was improved when considering simultaneously biological and radiological markers obtained on ICU admission (AUC = 0.78).

**Conclusions:**

The extent of lung parenchyma lesions on CT was associated with inflammation, and the combination of coagulation and inflammatory biomarkers and the extent of the lesions predicted the poorest outcomes.

## Introduction

Almost all patients admitted in ICU for severe acute respiratory syndrome Coronavirus 2 (SARS-CoV-2) got a chest computed tomography (CT) on hospital and/or ICU admission [1]. The sensitivity of CT-scans is close to 90% for confirming the SARS-CoV-2 aetiology of acute respiratory distress syndrome (ARDS). Various radiological presentations, such as ground-glass opacities, vascular enlargements, air bronchograms, and parenchymal condensations, are commonly observed, often without direct correlation to initial clinical symptoms. The extent and categorization of lung parenchymal lesions on chest CT scans can offer prognostic insights, identifying patients at risk of severe forms of SARS-CoV-2 pneumonia, intensive care unit (ICU) admission, or hospital mortality [2–6]. The advent of artificial intelligence (AI) software for assessing these lesions on CT scans represents a significant advance in reliability and reproducibility. Few studies have explored the prognostic value of AI tools in ICU patients with severe SARS-CoV-2 pneumonia [7–9]. Beyond imaging, various prognostic criteria on ICU admission include older age, male sex, diabetes, obesity, high blood pressure, immunosuppression, severity of hypoxemia, lymphopenia[10–13], elevated plasma biomarkers of non-specific inflammation such as C-reactive protein (CRP) and ferritin levels [10–12, 14, 15], and of specific inflammation cytokines such as interleukin 1 (IL-1)) [10–12, 15], interleukin 6 (IL-6) [10, 11, 16, 17], and standard coagulation test results indicative of hypercoagulability [18]. Despite therapeutic advancements, such as the proven efficacy of steroids [13], inconsistencies in results for other anti-inflammatory treatments, including IL-6 receptor antagonists [19] and antiviral therapies [20], have raised concerns about patient heterogeneity.

This study aims therefore to investigate the epidemiological and biological characteristics of ICU-admitted SARS-CoV-2 pneumonia patients, the extent of their lung lesions as determined by AI on chest CT scans, and their outcomes. We also give a description of the cohort according to the extent of lung parenchymal lesions.

## Materials and methods

### Data source

This is a retrospective, monocentric cohort study based on prospectively recorded data. The study was conducted in the Medical Intensive Care Unit (ICU) at Clermont-Ferrand University Hospital in France, from 1^st^ February 2020, to 30^th^ June, 2021. Comprehensive and clear information was provided to all patients or their representatives, and written informed consent was obtained for the storage and research use of residual blood samples collected as part of routine care (IRB No. 20.03.20.56342 from CPP-Ile-de-France VI Groupe Hospitalier Pitié-Salpetriêre). All patients were included in the OutcomeRea^TM^ database according to detailed data collection methods and quality standards specified elsewhere [21]. The OutcomeRea^TM^ database, in compliance with French law, received approval from the French Advisory Committee for Data Processing in Health Research (CCTIRS) and the French Informatics and Liberty Commission (CNIL, registration no. 8999262). The database protocol was submitted to the Institutional Review Board of Clermont-Ferrand University Hospital, which waived the need for informed consent (CECIC Clermont-Ferrand -IRB n°5891; Ref: 2007–16). All the data of the patients were collected prospectively into the database and were anonymized. Access of the data for that study was achieved the 1st December 2022. The authors didn’t have access to information that could identify individual participants during or after data collection. All procedures were conducted in accordance with the ethical standards of the responsible committee on human experimentation, whether institutional or regional, and in agreement with the Helsinki Declaration of 1975.

### Study population

Eligible participants were individuals aged over 18 with severe COVID-19, confirmed by a positive SARSCoV-2 test using RT-PCR. None of the patients were selected specifically on the basis of lung lesions. However, lung lesions per se help in the diagnosis of COVID-19 and therefore influenced the inclusion of patients in the study.

Exclusions were applied for patients with incomplete ICU follow-up before 30 June, 2021, referral from another ICU, absence of an analyzable chest CT-scan on ICU admission (+/- 48h), a decision to discontinue life-sustaining treatments within the first two days post-ICU admission, and an ICU length of stay (LOS) less than 72 hours.

### Data collection

Prospective data collection included demographics, comorbidities, baseline severity indices, such as the Simplified Acute Physiology Score II (SAPS II) [22] and the Sequential Organ Failure Assessment (SOFA) [23] score, treatments, and laboratory features on ICU admission such as blood count, serum BNP, plasma coagulation biomarkers (D-dimers and fibrinogen levels), serum inflammatory biomarkers (CRP, ferritin, IL-1, IL-6, IL-10, procalcitonin [PCT]), and human histocompatibility leukocyte antigen expression rate on blood monocytes (mHLA-DR). In order to assess the balance between inflammation and immunomodulation, we also studied the IL-6/mHLA-DRx10^3^ parameters as reported by Bonnet et al. [24].

Patients were categorized as being chronically immunosuppressed if they presented the following characteristics: aplasia (lymphocytes < 1000/mm3), corticosteroid treatment (if treatment duration >1 month or if dosage was >2mg/kg regardless of duration), HIV (positive serology), and AIDS.

Throughout the ICU stay, variables encompassed clinical and biological aspects, ventilatory and organ support, nosocomial infections, and outcomes (ICU and hospital LOS, ventilatory and oxygen-free days [VFD and OFD] at day 60, vital status at ICU and hospital discharge, and at day 60).

Chest CT-scans were performed on ICU admission (+/- 48h) on a 64-strip scanner from General Electric® (Milwaukee, USA). The percentage of parenchymal lesions was assessed with artificial intelligence (AI) software (Thoracic VCAR software, GE Healthcare, Schedule 1) [25]. This post-processing software allows the use of an automatic threshing method based on density measurements in Hounsfield Units (HU): a density threshold higher than -686 HU thus made it possible to differentiate and quantify the condensation or frosted glass zones related to Covid 19 lung parenchyma. It has the advantage of allowing a reproducible standardization of the measurement [26]. No perfusion alterations were quantified, which would have required a specific dual energy scan. All the extents of lung lesions determined by IA were validated and/or modified, if necessary, by an expert radiologist with either 20 years’ (LC) or 5 years’ (RCdB) experience.

Each patient was then classified according to the extent of lung parenchymal lesions (< 25%, 26–50%, 51–75%, ≥75%) [25].

### Statistical analysis

Baseline characteristics were reported as median [interquartile range] and n (%) for quantitative and qualitative variables, respectively. Quantitative variables were compared with nonparametric tests, the Mann-Whitney test or the Kruskal-Wallis test, as appropriate. Qualitative variables were compared with Pearson’s Chi-square test or Fisher’s exact test, as appropriate. Continuous variables were dichotomized according to their median observed in our dataset before imputation if necessary.

Correlations between variables were explored by Pearson correlation coefficient and representation using a correlation matrix.

We described and compared variables (baseline characteristics, laboratory features, radiological findings and outcomes) within and between categories defined by the extent of lung parenchymal lesions. Prediction of day-60 death by biological features and/or extent of the lesions were determined by their AUC of the ROC curves. The DeLong method was used to compare all the AUCs [27].

Risk factors for death at day 60 were investigated using uni- and then multivariate Cox survival models. The proportionality of the risks was carefully checked. Variables with a p-value < 0.1 in univariate analysis were tested for in a multivariate model. A backward selection with only variables having a p-value < 0.05 were made to determine the final multivariate model. The results were expressed in hazard ratio with their 95% confidence intervals.

Using univariate logistic regression and then a random forest approach, we determined the variables associated with an extent of the lesions on CT-scan of at least 75%. The random forest method is a type of decision-tree learning algorithm that is able to address nonlinear relationships and complex interactions between potential explanatory variables and to rank the relative importance of each factor in predicting the membership of a category. It was conducted using the randomForest package in R. A p-value lower than 0.05 was considered significant. All statistical analyses were performed with SAS software, Version 9.4 (SAS Institute, Cary, NC) and R (version 3.6.3). The sample size of our cohort was not prospectively determined, and we included all the patients who had inclusion criteria during the period of recruitment.

## Results

### Subject demographics

**Fig 1:**
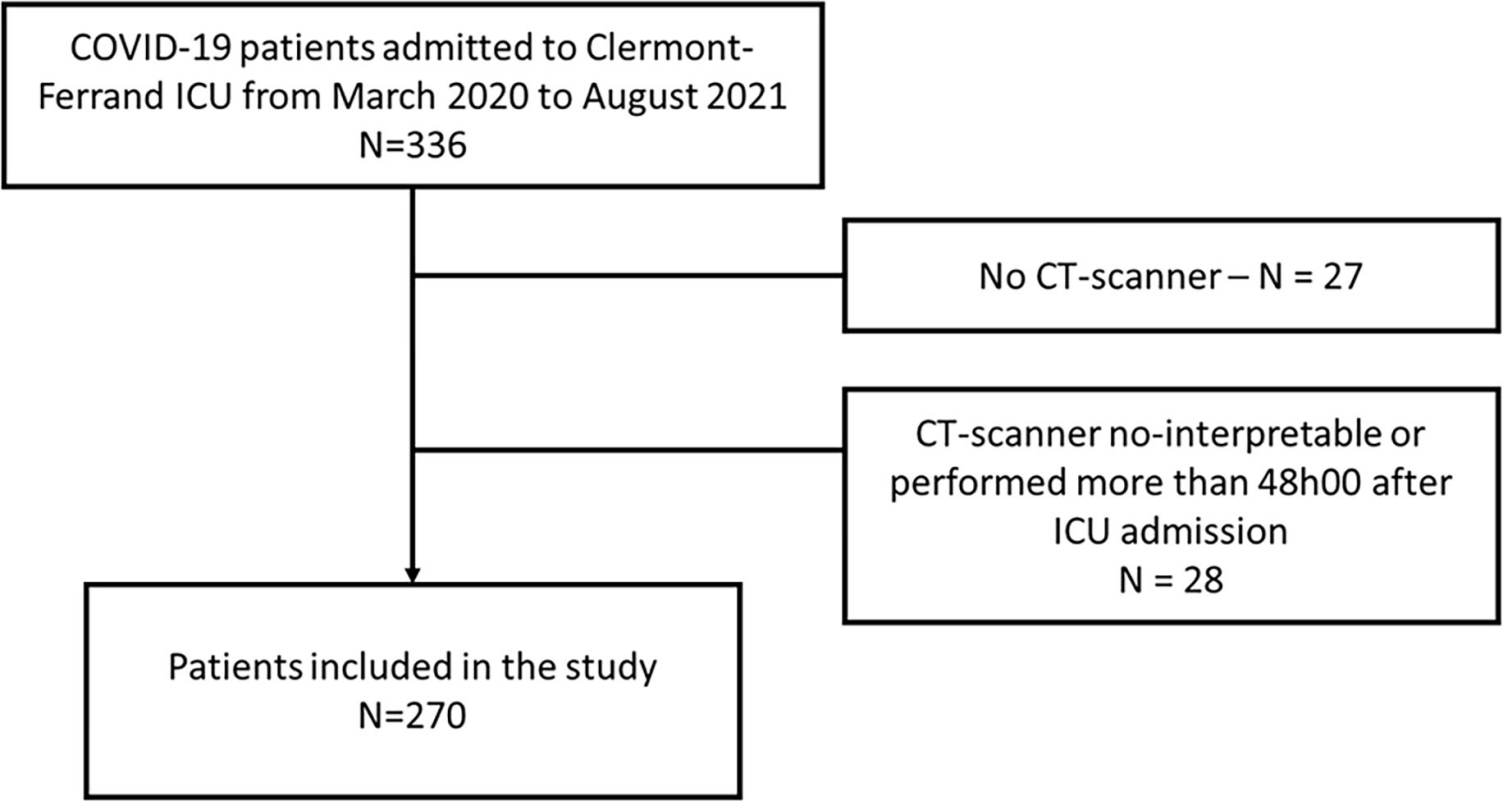
Flow chart. ICU: Intensive care unit.

Demographic details of the study cohort were recorded for 270 out of 325 critically ill COVID patients admitted to the medical ICU of Clermont-Ferrand hospital during the study period (Fig 1). Of these, 71.5% were male, with a median age of 68.8 years and a median BMI of 28.8 kg/m^2^. Predominant coexisting conditions included obesity and cardiovascular disease. The median duration from symptom onset to ICU admission was 9 days, and from hospital to ICU admission, 2 days. Upon ICU admission, the median SAPS II was 36, SOFA score was 5, and the PaO2/FiO2 ratio was 116 mmHg. Only 11.5% of patients were intubated on admission. Lymphocyte count, plasma CRP level, and plasma fibrinogen level on admission had median values of 0.7 G/L, 113.5 mg/L, and 7.2 g/l, respectively. During ICU stay, 33.3% of patients were eventually intubated: they had a median ICU length of stay of 8 days and a hospital mortality rate of 30.7% (S1 Table and Table 1).

**Table 1:** Characteristics of the whole population and depending on the extent of the lung parenchymal lesions.

N(%) or median [IQR]	All	< 25%	25% - 50%	50% - 75%	≥ 75%	p-value
**Number of patients**	270	49	99	90	32	.
**Percentage of lung parenchymal lesions (%)**	46.9 [30; 61]	18 [10; 21]	38 [31; 45]	60 [54; 65]	77 [75; 82]	<0.01
**Time from 1**^**st**^ **symptoms to CT-scan, days**	8 [5; 11]	7.5[3; 10.5]	8[5; 10]	8[6; 11]	10[8; 16]	0.02
**Time from 1**^**st**^ **symptoms to ICU admission, days**	9 [7; 11]	9 [6; 11]	9 [6; 11]	9 [7; 11]	10 [8; 14]	0.62
**Time from ICU admission to CT scan, days**	1[1; 3]	1[0; 3]	1[0; 2]	0[0; 1]	0[0; 1]	<0.01
**Before August 2020 (1st Wave)**	16 (5.9)	6 (12.24)	3 (3.03)	7 (7.78)	12 (37.5)	0.12
**Age, years**	68.8 [60.5; 74.8]	71.1 [66.2; 76.4]	67.6 [59.8; 73.8]	68.6 [60.0; 75.9]	69.3 [59.1; 75.7]	0.10
**Sex (Male)**	193 (71.5)	34 (69.39)	78 (78.79)	58 (64.44)	23 (71.88)	0.18
**BMI > 30 (kg/m^2^)**	119 (44.1)	17 (34.69)	43 (43.43)	43 (47.78)	16 (50)	0.44
**Comorbidities**						
**Cardiovascular disease**	53 (19.6)	15 (30.61)	19 (19.19)	12 (13.33)	7 (21.88)	0.11
**Chronic respiratory disease**	20 (7.4)	4 (8.16)	7 (7.07)	8 (8.89)	1 (3.13)	0.75
**Chronic renal disease**	21 (7.8)	5 (10.2)	8 (8.08)	5 (5.56)	3 (9.38)	0.77
**Immunosuppression§**	44 (16.3)	11 (22.45)	18 (18.18)	12 (13.33)	3 (9.38)	0.35
**Time from hospital to ICU admission, days**	2 [1; 5]	3 [2; 6]	2 [1; 5]	2 [1; 4]	2 [1; 7]	0.04
**On admission**						
**SAPS II**	36 [29; 45]	37 [32; 48]	35 [29; 45]	36 [31; 42]	35 [28.5; 47]	0.37
**SOFA score**	5 [3; 6]	4 [3; 5]	4 [3; 6]	5 [4; 7]	5 [4; 7.5]	0.09
**SOFA score without respiratory item**	1 [1; 3]	1 [1; 3]	1 [0; 3]	1 [0; 3]	2 [1; 4.5]	0.31
**SOFA score respiratory item > 2**	193 (71.5)	29 (59.18)	71 (71.72)	68 (75.56)	25 (78.13)	0.17
**SOFA score cardio-vascular > 2**	34 (12.6)	2 (4.08)	11 (11.11)	14 (15.56)	7 (21.88)	0.08
**SOFA score kidney > 2**	37 (13.7)	9 (18.37)	15 (15.15)	10 (11.11)	3 (9.38)	0.55
**PaO2/FiO2**	116 [77.1; 168.9]	124 [95.4; 194.3]	122 [78.; 8 200]	115.9 [76.3; 164]	90 [62.5; 129]	0.03
**Invasive mechanical ventilation**	31 (11.5)	3 (6.12)	9 (9.09)	13 (14.44)	6 (18.75)	0.22
**High-flow nasal cannula**	168 (62.2)	28 (57.14)	64 (64.65)	58 (64.44)	18 (56.25)	0.69
**Vasopressors**	34 (12.6)	2 (4.08)	11 (11.11)	14 (15.56)	7 (21.88)	0.08
**Renal replacement therapy**	10 (3.7)	2 (4.08)	3 (3.03)	3 (3.33)	2 (6.25)	0.86
**Steroids**	227 (84.1)	38 (77.55)	85 (85.86)	75 (83.33)	29 (90.63)	0.42
**Laboratory features**						
**Neutrophils(G/L)**	6.9 [4.8; 9.9]	6.3 [4.76; 9.32]	6.71 [5.07; 9.52]	6.82 [4.56; 9.8]	10.19 [5.81; 12.36]	0.10
**Lymphocytes (G/L)**	0.7 [0.5; 1.1]	0.64 [0.44; 1.06]	0.77 [0.52; 1.08]	0.73 [0.49; 1.19]	0.87 [0.51; 1.27]	0.51
**Monocytes (G/L)**	0.4 [0.2; 0.6]	0.4 [0.2; 0.59]	0.41 [0.24; 0.62]	0.41 [0.23; 0.54]	0.45 [0.26; 0.74]	0.67
**Hematocrit (%)**	39 [35; 42]	39 [35; 43]	40 [36; 42]	38.5 [36; 42]	38.5 [34; 42]	0.90
**Platelets (g/l)**	245.5 [197; 309]	238 [198; 302.64]	252 [194; 307]	245 [198; 325]	243 [192; 292]	0.94
**Prothrombin time, %**	87 [76; 93]	86 [78; 93]	89 [78; 97]	84.5 [75; 91]	87.5 [81; 91.34]	0.09
**D-dimer (ng/mL)**	1251.5 [786; 2246]	1100 [701; 1775]	1215 [795; 1928]	1505.5 [820; 3345]	1240 [946; 2927.5]	0.03
**Fibrinogen (g/L)**	7.2 [6.3; 7.9]	6.8 [6; 7.4]	7.2 [6.6; 8]	7.2 [6; 8]	7.55 [6.85; 8.4]	0.03
**Creatininemia (umol/L)**	84 [67; 112]	89 [61; 125]	80 [69; 104]	79.5 [64; 112]	91.5 [65; 124]	0.62
**Bilirubinemia (umol/L)**	10 [7; 13.7]	10 [6.84; 14]	9 [6.84; 13.68]	10.13 [7; 13.68]	10.63 [9; 13]	0.38
**Lactate (mmol/L)**	1.4 [1.1; 1.8]	1.4 [1.1; 1.7]	1.4 [1.2; 1.8]	1.4 [1.1; 1.9]	1.55 [1.3; 1.8]	0.40
**Procalcitonin (ug/L)**	0.3 [0.1; 0.9]	0.28 [0.11; 0.96]	0.26 [0.13; 1.06]	0.27 [0.16; 0.74]	0.4 [0.17; 0.96]	0.72
**C-reactive protein (mg/L)**	130.6 [81.9; 184]	125 [76.3; 171]	128 [81.2; 175]	133.7 [76.1; 183.4]	185 [112.5; 238.6]	0.03
**Ferritin (ug/L)**	1217 [690; 2135]	1209 [749; 2187]	1538 [780; 2152]	1123.5 [635; 1976]	935 [501.5; 1547]	0.13
**IL-10 (pg/mL)**	4.7 [2.5; 9]	4.26 [1.9; 8.3]	4.9 [2; 9.5]	5.35 [2.9; 9.4]	3.9 [2.31; 6.63]	0.44
**IL-1b (pg/mL)**	0 [0; 1.1]	0.1 [0; 1]	0.2 [0; 1.4]	0 [0; 0.85]	0 [0; 0.81]	0.27
**IL-6 (pg/mL)**	45.7 [16.6; 101]	44.5 [18.5; 87.6]	49.1 [16.3; 110]	49.4 [16.3; 143.9]	38.4 [17.9; 68.2]	0.67
**mHLA DR (pg/mL)**	7762.5 [4293; 12397]	8154 [4708; 12335]	7943 [4228; 13330]	7897 [3887; 11819]	6386.5 [4622; 10830.5]	0.77
**IL-6/mHLA DR x 1000**	5.6 [1.6; 13.6]	5.4 [1.94; 11.8]	6.05 [1.33; 13.63]	5.12 [1.72; 20.19]	6.26 [3.09; 10.38]	0.95
**During ICU stay**						
**Vasopressors, n (%)**	90 (33.3)	10 (20.41)	28 (28.28)	35 (38.89)	17 (53.13)	<0.01
**Invasive mechanical ventilation, n (%)**	90 (33.3)	11 (22.45)	30 (30.3)	33 (36.67)	16 (50)	0.06
**Renal replacement therapy, n (%)**	46 (17)	7 (14.29)	14 (14.14)	18 (20)	7 (21.88)	0.59
**Pulmonary embolism, n (%)**	14 (5.2)	2 (4.08)	5 (5.15)	6 (6.67)	1 (3.13)	0.85
**Ventilator-associated pneumonia, n (%)**	35 (13.1)	1 (2.04)	6 (6.19)	21 (23.33)	7 (21.88)	<0.01
**Invasive aspergillosis, n (%)**	13 (4.9)	0	6 (6.19)	5 (5.56)	2 (6.25)	0.38
**Ventilatory-free days at day 60**	60 [0; 60]	60 [0; 60]	60 [0; 60]	60 [0; 60]	0 [0; 60]	0.01
**ICU length of stay, days**	8 [5; 13]	7 [4; 11]	7 [4; 10]	8 [5; 15]	9 [5; 14]	0.08
**Hospital length of stay, days**	14.5 [10; 24]	15 [8; 25]	14 [9; 20]	16.5 [11; 27]	13.5 [9.5; 27.5]	0.36
**Hospital death**	83 (30.7)	14 (28.57)	23 (23.23)	30 (33.33)	16 (50)	0.04
**Day-60 mortality**	92 (34.1)	15 (30.61)	25 (25.25)	35 (38.89)	17 (53.13)	0.02

BMI: Body mass index; SAPS II: Simplified acute physiology score II; SOFA: Sequential organ failure assessment; Il: Interleukin; ICU: Intensive care unit

§Aplasia (lymphocytes < 1000/mm3); or corticosteroids (if treatment duration >1 month or if treatment amount >2mg/kg regardless of duration); or HIV (positive serology); AIDS (positive HIV serology and clinical complications: pneumocystis pneumonia, Kaposi’s sarcoma, tuberculosis, toxoplasmosis

### Description of the cohort according to the extent of lung parenchymal lesions

The four sub-groups defined by the extent of lung parenchymal lesions on chest CT scan of < 25%, 26–50%, 51–75%, and ≥ 75% comprised 49, 99, 90 and 32 patients, respectively (Table 1). The subgroups did not differ in terms of age, BMI, sex, comorbidities, SAPS II, serum levels of ferritin and cytokine (IL-1, IL-6 and IL-10), and quantitative expression of mHLA-DR. There were significant differences between the four groups in CRP levels (p = 0.03), time from hospital to ICU admission (p = 0.01), and the PaO2/FiO2 ratio (p<0.01). Patients with more extensive lung parenchymal lesions (≥ 75%) on admission had higher CRP serum levels and lower PaO2/FiO2 ratios. During ICU stay, the patients with more extensive lung parenchymal lesions on admission received more frequently invasive mechanical ventilation (p<0.01) and vasopressors (p = 0.01), developed more often nosocomial infections including VAP (p<0.01), had lower VFD at day 28 (p = 0.04) and day 60 (p = 0.01), and a higher hospital mortality rate (p = 0.02) and day-60 mortality rate (p = 0.01) (Table 2).

**Table 2:** Factors associated with lung parenchymal lesions > 75%, univariate analyses, logistic regression.

Variables	Vol < 75% (n = 235)	Vol≥75% (n = 35)	OR	ORIC	Pvalue
**Admission during the first wave**	16 (6.7)	0	-	-	-
**Time from first symptoms to ICU admission > 10 days**	82 (35)	15 (42.9)	1.39	[0.68; 2.86]	0.37
**Time from hospital to ICU admission > 4 days**	61 (26.1)	13 (37.1)	1.68	[0.8; 3.53]	0.17
**Age > 65 years**	105 (44.7)	18 (51.4)	1.31	[0.64; 2.67]	0.46
**Sex (male)**	167 (71.4)	25 (71.4)	1.00	[0.46; 2.2]	0.99
**Body mass index > 30 kg/m^2^**	103 (44)	16 (45.7)	1.07	[0.52; 2.19]	0.85
**Comorbidities**	120 (51.1)	17 (48.6)	0.91	[0.44; 1.84]	0.78
**Immunosupression**	41 (17.2)	3 (9.4)	0.50	[0.14 ; 1.71]	0.27
**SOFA respiratory item > 2**	166 (70.9)	27 (77.1)	1.38	[0.6; 3.2]	0.45
**SOFA cardio-vascular item > 2**	26 (11.1)	7 (20)	2.00	[0.79; 5.03]	0.14
**SOFA kidney item > 2**	34 (14.5)	3 (8.6)	0.55	[0.16; 1.9]	0.35
**Invasive mechanical ventilation**	25 (10.7)	6 (17.1)	1.72	[0.65; 4.55]	0.27
**Corticosteroids**	194 (82.9)	31 (88.6)	1.60	[0.53; 4.78]	0.40
**Neutrophils > 6.5 G/L**	130 (55.3)	26 (74.3)	2.33	[1.05; 5.2]	0.04
**Lymphocytes < 0.7 G/L**	115 (48.9)	16 (45.7)	0.88	[0.43; 1.79]	0.72
**Monocytes > 0.4 G/L**	117 (49.8)	22 (62.9)	1.71	[0.82; 3.55]	0.15
**D-dimers > 1500 ng/mL**	91 (38.7)	14 (40)	1.05	[0.51; 2.18]	0.89
**Fibrinogen > 8 g/L**	43 (18.3)	9 (25.7)	1.55	[0.68; 3.53]	0.30
**Lactate > 2 mmol/L**	43 (18.3)	6 (17.1)	0.92	[0.36; 2.36]	0.87
**Procalcitonin > 0.5pg/mL**	80 (34)	14 (40)	1.29	[0.62; 2.68]	0.49
**C-reactive protein > 100mg/L**	141 (60)	29 (82.9)	3.22	[1.29; 8.06]	0.01
**Ferritin > 1000ug/L**	146 (62.1)	18 (51.4)	0.65	[0.32; 1.32]	0.23
**IL-10 > 4.5 pg/mL**	123 (52.3)	17 (48.6)	0.86	[0.42; 1.75]	0.68
**IL-1b > 0.2pg/mL**	106 (45.1)	14 (40)	0.81	[0.39; 1.67]	0.57
**IL-6 > 45 pg/mL**	121 (51.5)	17 (48.6)	0.89	[0.44; 1.81]	0.75
**mHLA-DR > 10000pg/mL**	93 (39.6)	9 (25.7)	0.53	[0.24; 1.18]	0.12
**IL-6/mHLA DR (x 1000)> 4.8**	118 (50.2)	21 (60)	1.49	[0.72; 3.06]	0.28

SOFA: Sequential organ failure assessment, IL: Interleukin, mHLA-DR: Human leukocyte antigen isotype DR

**Fig 2:**
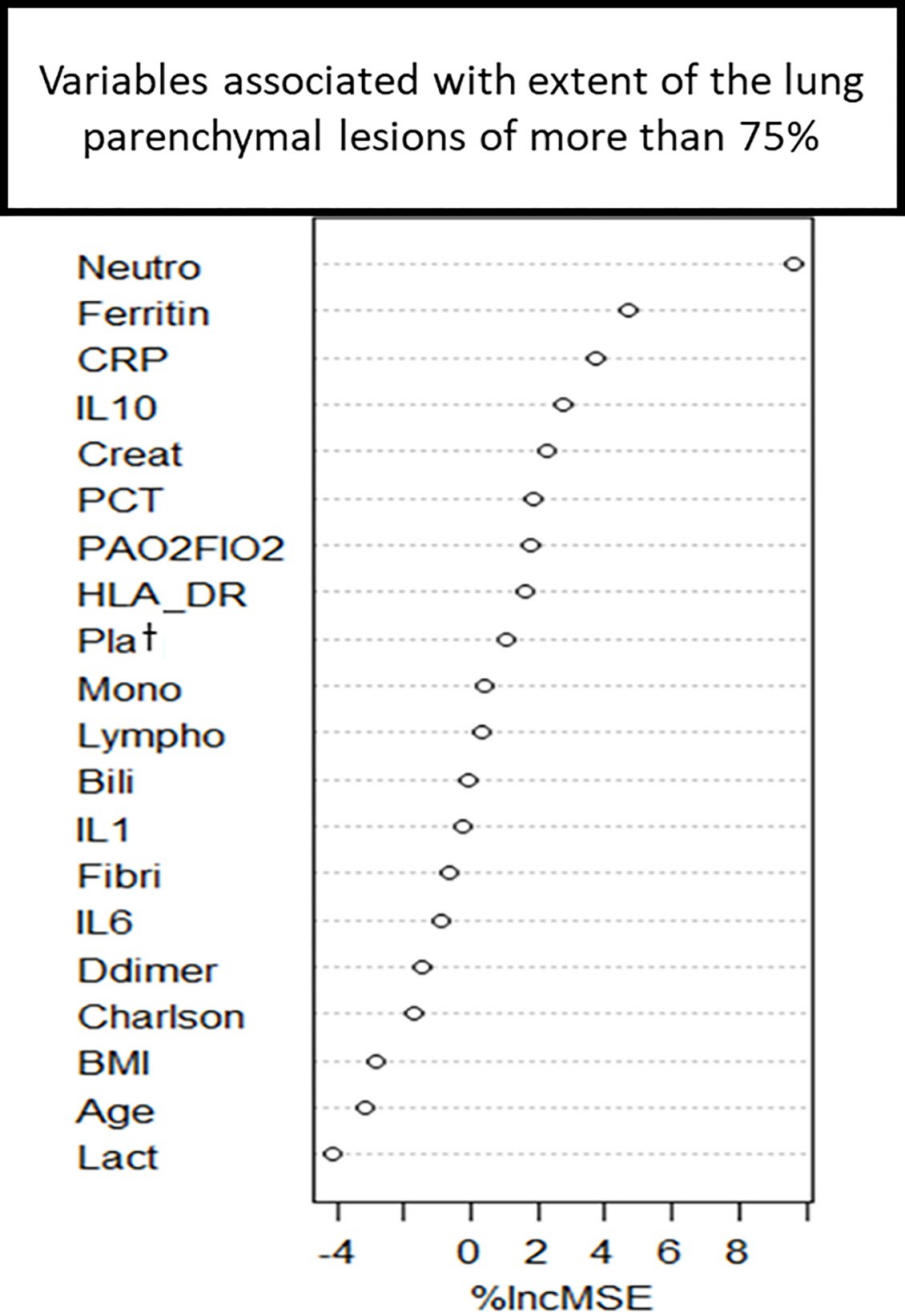
Factors associated with the extent of lung lesion of more than 75% on CT scanner determined by random forest. Neutro: Neutrophils, Ferritin: ferritinemia, CRP: C-reactive protein, IL: Interleukin, Create: Creatininemia, PCT: Procalcitonin; HLA DR: HLA-DR monocytic–expression rate, Plat: Platelet, Mono: Monocytes, Fibri: Fibrinogen, BMI: Body mass index; Lact: Lactate.

**Fig 3:**
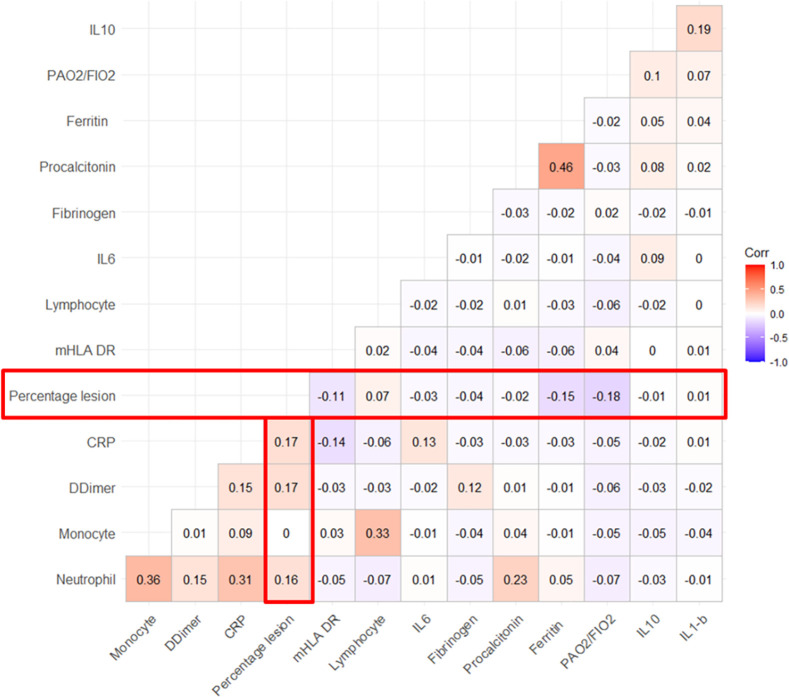
Correlation between laboratory parameters and the extent of the lung lesions on CT scan on ICU admission. Ferritin: ferritinemia, CRP: C-reactive protein, IL: Interleukin, mHLA DR: HLA-DR monocytic–expression rate.

The factors associated with an extent of lung lesion of more than 75% identified by random forest method and by logistic regression are given in Fig 2 and Table 2 respectively. Inflammation biomarkers including the levels of neutrophils, CRP, ferritin and Il10 seemed to be the indices the most associated with the severity of the extent of the lung lesions. However, no strong correlations with the extent of lung lesion and inflammatory and coagulation biomarkers were observed on ICU admission (Fig 3). Of note, compared to patients without immunosuppression, patients with immunosuppression on admission tended to have lower extent of their lung lesions, and higher Il-6 levels, but similar mHLA-DR levels (S2 Table).

### Risk factors of death

**Fig 4:**
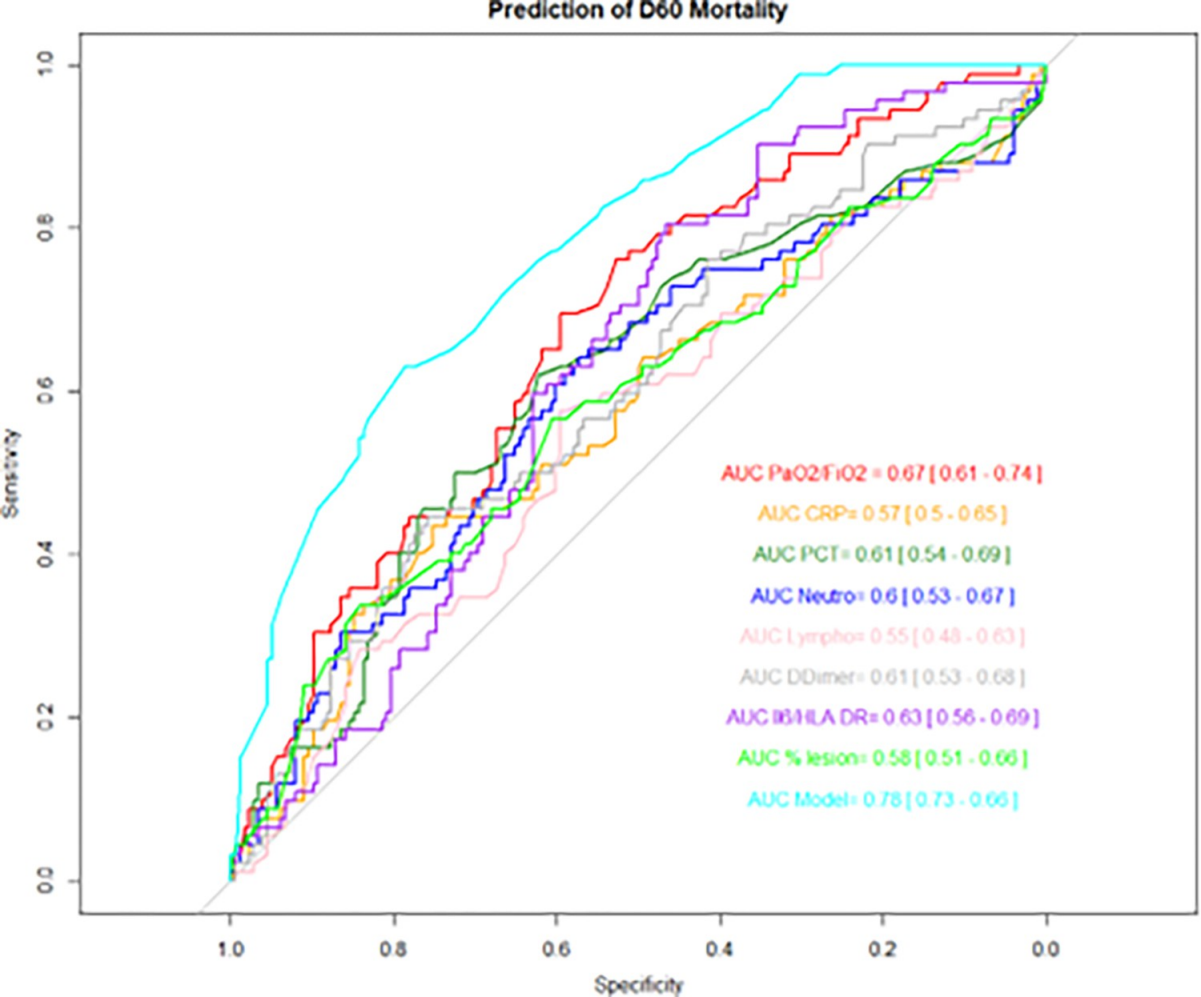
Prediction of D60 mortality depending on laboratory parameters, the extent of the lung lesions on CT scan and model of prediction on ICU admission. Model = Age+ SOFA respi + Lymphocytes, + PCT + Il6/mHLA-DR + % the extent of lung lesion, according to the results Table 3. SOFA: Sequential organ failure assessment, PCT: Procalcitonin, Il: Interleukin, mHLA DR: HLA-DR monocytic–expression rate.

In univariate and multivariate analyses (Table 3), an extent of lung lesions of more than 75% was independently associated with day-60 mortality (aHR = 1.72[1.06; 2.78], p = 0.03). The prediction of death at day 60 was improved when considering simultaneously biological and radiological markers obtained on ICU admission (AUC = 0.78). The ratio Il6/mHLA and PaO2/FiO2 showed moderate performances with AUCs of 0.63 and 0.67, respectively, and the extent of lung involvement was only mild (0.58 [0.51–0.66]) and not statistically better than those provided by biological parameters. (Fig 4 and S3 Table).

**Table 3:** Risk factors of death at day 60, univariate analyses, Cox survival models.

Variables, n (%)	Alive	Dead	HR	IC 95% HR	p-value	HR	IC 95% HR	p-value
**Number of patients**	178	92						
**Admission during the first wave**	10 (5.6)	6 (6.5)	0.88	[0.38; 2.01]	0.76			
**Time from first symptoms to ICU admission > 10 days**	73 (41.2)	24 (26.1)	0.64	[0.4; 1.02]	0.06			
**Age, years**	62 (34.8)	61 (66.3)	2.21	[1.43; 3.41]	<0.01	2.46	[1.59; 3.78]	<0.01
**Sex (male)**	128 (72.3)	64 (69.6)	0.94	[0.6; 1.47]	0.80			
**Body mass index > 30 kg/m^2^**	80 (45.2)	39 (42.4)	0.78	[0.51; 1.18]	0.23			
**Comorbidities**	80 (44.9)	57 (62)	1.36	[0.89; 2.07]	0.15			
**Immunosuppression**	24 (13.6)	20 (21.7)	1.49	[0.91; 2.46]	0.11			
**SOFA respiratory item > 2**	116 (65.5)	77 (83.7)	1.94	[1.12; 3.37]	0.02	1.73	[1.03; 2.92]	0.04
**SOFA cardio-vascular item > 2**	13 (7.3)	20 (21.7)	1.89	[1.15; 3.1]	0.01			
**SOFA kidney item > 2**	21 (11.9)	16 (17.4)	1.17	[0.68; 2]	0.58			
**Invasive mechanical ventilation**	13 (7.4)	18 (19.6)	1.53	[0.92; 2.57]	0.10			
**Corticosteroids**	146 (82.5)	79 (85.9)	1.62	[0.9; 2.93]	0.11			
**Neutrophils > 6.5 G/L**	92 (51.7)	64 (69.6)	1.59	[1.02; 2.49]	0.04			
**Lymphocytes < 0.7 G/L**	77 (43.3)	54 (58.7)	1.49	[0.98; 2.25]	0.06	1.61	[1.06; 2.46]	0.03
**Monocytes > 0.4 G/L**	89 (50)	50 (54.3)	1.12	[0.74; 1.68]	0.60			
**D-dimers > 1500 ng/mL**	61 (34.3)	44 (47.8)	1.59	[1.06; 2.39]	0.03			
**Fibrinogen > 8 g/L**	35 (19.7)	17 (18.5)	1.10	[0.65; 1.86]	0.73			
**Lactate > 2 mmol/L**	26 (14.6)	23 (25)	1.60	[1; 2.56]	0.05			
**Procalcitonin > 0.5 pg/mL**	49 (27.5)	45 (48.9)	1.88	[1.25; 2.84]	<0.01	2.15	[1.41; 3.30]	<0.01
**C-reactive protein > 100mg/L**	109 (61.2)	61 (66.3)	1.15	[0.75; 1.78]	0.52			
**Ferritin > 1000ug/L**	108 (60.7)	56 (60.9)	0.98	[0.64; 1.49]	0.92			
**IL-10 > 4pg/mL**	74 (41.6)	66 (71.7)	2.71	[1.72; 4.27]	<0.01			
**IL-1b > 0.2pg/mL**	76 (42.7)	44 (47.8)	1.14	[0.76; 1.72]	0.53			
**IL-6 > 40pg/mL**	81 (45.5)	57 (62)	1.34	[0.88; 2.04]	0.18			
**mHLA-DR > 10000pg/mL**	72 (40.4)	30 (32.6)	0.87	[0.56; 1.35]	0.54			
**IL-6/mHLA DR (x 1000)> 4.8**	76 (42.7)	63 (68.5)	2.14	[1.37; 3.33]	<0.01	2.17	[1.41; 3.45]	<0.01
**% lung parenchymal lesions ≥ 75%**	17 (9.6)	18 (19.6)	1.84	[1.1; 3.09]	0.02	1.72	[1.06; 2.78]	0.03

SOFA: Sequential organ failure assessment, IL: Interleukin, mHLA-DR: Human leukocyte antigen isotype DR

## Discussion

This investigation showed a direct correlation between the severity of lung parenchymal lesions upon ICU admission and the severity of illness in patients with SARS-CoV2 pneumonia. The association was evidenced by more profound hypoxemia, increased vasopressor requirements, elevated rates of ventilator associated pneumonia (VAP) and mortality, and fewer ventilatory-free days (VFD). Similar findings have been reported by other researchers. For instance, Colombi et al. [28] found that COVID-19 patients with extensive lung lesions on CT scan were more likely to be admitted to the ICU or face mortality. Other studies related parenchymal condensations [29] and pleural effusions to poorer prognosis [30]. Extensive lung lesions, irrespective of radiological image type, were consistently associated with higher mortality rates [9, 31, 32].

In our study, only CRP and neutrophils seemed to be higher for patients with the greatest extent of the lung lesions. The association between systemic inflammation and extension of lung lesions in COVID-19 has been scantily documented [33]. Wu et al. [34] reported that the levels of lymphocytes and CRP differed between patients according to the severity of lung lesions as defined by their radiological score (RadScore). Similarly, the radiological score proposed by Szabo et al. [35] was also associated with absolute leukocyte and platelet counts, CRP, PCT, LDH, D-dimer, ferritin, IL-6, and negatively correlated with lymphocyte count.

We then showed that most of the inflammatory biomarkers, including cytokines and mHLA-DR, were associated with survival. We also confirmed the value of the IL-6/mHLA-DR ratio as reported by Bonnet et al., which proved useful in identifying the most severe patients and independently predicting an increased risk of death [24]. During sepsis, immunoparalysis [36] is generally associated with an increased risk of nosocomial infections and mortality [37, 38] and has already been investigated in COVID-19. For instance, Spinetti et al. showed that patients admitted to the ICU for severe SARS-CoV2 pneumonia had marked lymphopenia and lowered mHLA-DR [39]. Bonnet et al showed that severe SARS-CoV2 pneumonia was characterized by the association of a moderate increase in systemic pro-inflammatory cytokines with severe monocyte downregulation [24]. It is also interesting to note that immunosuppressed patients tended to have a lesser extent of lung lesions but higher Il6 levels and similar mHLA DR levels on ICU admission. Assessment of immunomodulation among previous immunocompromised patients warrants further research.

We also observed that the combination of inflammatory biomarkers and radiological findings greatly improve prediction of day-60 mortality. Some studies also assessed the combined performances of radiological findings and biomarkers to predict COVID-19 outcomes [9, 40]. For instance, the RadScore proposed by Wu et al. [34] was equally reliable as their proposed clinical score, and prognostication was improved when both clinical and radiological parameters were taken together. In the study of Cappabianca [9, 41] involving 103 patients hospitalized for COVID-19, the authors reported that the best prediction of prognosis was obtained by combining clinical, laboratory, and CT findings. In their study, the final model for prediction of admission to the ICU and/or death comprised older age, a low SpO2, high alanine aminotransferase (ALT) level, increased emphysema volume, low residual healthy lung parenchyma volume, and high consolidation volume.

Finally, we used CT AI software to determine the extent of lesion of the lung. The advantages of quantitative CT AI software under radiologist supervision include a significant reduction in interpretation time, fast learning curve, and increased objectivity of the quantitative severity assessment of the affected lung parenchyma, which decreases variability between readers [25]. Other authors have also used quantitative CT AI software and reported good feasibility, and interesting results for prognostication [40, 42]. However, the AI software had certain limitations. First, Thoracic VCAR® is not CE-marked for lung study for COVID-19. Second, a great variability among quantitative measurements provided by computer tools have already been reported and therefore our results should be interpreted cautiously. For instance, in the seminal study of Grassi et al. [25], the radiological severity score was only moderately correlated with the residual healthy lung parenchyma and ground-glass opacity volume obtained by Thoracic VCAR® software. Third, the AI software did not determine the different patterns of the CT lesions, and we did not record them either. Hence, in our analyses we only took into account the extent of the lesions. Fourth, it has also been reported that Thoracic VCAR software is not able to perform volume segmentation in certain cases, underlining the limitations of IA-based algorithm software [25].

This study has both advantages and limitations. Its main strength is the quality of the data collected prospectively, including inflammatory cytokine and mHLA DR levels. It is one of the first studies focusing on patients with SARS-CoV2 pneumonia admitted to the ICU to report the extent of lung parenchymal lesions using reliable and reproducible AI software (Thoracic VCAR software, GE Healthcare).

Our study has several limitations the first of which are its single-centre design and its retrospective nature. Second, a four-grade scale was used to characterize the extent of lung lesions whereas other authors proposed a scale with five levels: none, mild (inferior or equal to 25%), moderate (26–50%), severe (51–75%) and critical (76–100%). In our cohort, only five patients had no lesions on CT-scan at ICU admission, and therefore we merged the levels none and mild. Third, we looked for the factors associated with an extent of lung lesion of more than 75%. This threshold seemed to us better able to identify the most severe patients. Fourth, the cut-offs used for inflammatory biomarkers were based on the median value observed in our datasets. Other cut- off points could have been taken into account. For instance, very recently, Monneret et al. proposed a cut-off point of 5 479 Ab/C in a cohort of critically ill patients to distinguish survivors from non-survivors [43]. In previous studies involving ICU patients a cut-off value of 8000 Ab/C has been more frequently used [44–46]. Fifth, some covariates such as vaccination status and variants were lacking in the presentation of our COVID-19 patients Finally, our results cannot be extended to most of the other pulmonary diseases, including bacteriological pneumonia and inflammatory pneumonia.

## Conclusion

The scientific content of the study gives crucial insights into the prognostic factors and outcomes of patients with severe SARS-CoV2 pneumonia admitted to the ICU. The correlation between the extent of lung parenchymal lesions, as analyzed with AI software, and the severity of the illness is a significant finding, supported by a comprehensive examination of clinical, radiological, and immunological parameters. The study underscores the importance of considering the pro-inflammatory/immunoparalysis balance, especially using the IL-6/mHLA-DR ratio, in predicting patient outcomes. Combined scores with clinical risk factors, biological signatures and CT-scan findings could be used by clinicians for decision- making during hospitalization to predict high-risk patients.

## Supporting information

S1 File (XLSX)

S1 Table Characteristics of the whole population before imputation. BMI: Body mass index; SAPS II: Simplified acute physiology score II; SOFA: Sequential organ failure assessment; Il: Interleukin; ICU: Intensive care unit. §Aplasia (lymphocytes < 1000/mm3); or corticosteroids (if treatment duration >1 month or if treatment amount >2mg/kg regardless of duration); or HIV (positive serology); AIDS (positive HIV serology and clinical complications: pneumocystis pneumonia, Kaposi’s sarcoma, tuberculosis, toxoplasmosis.
(DOCX)

S2 Table Comparisons of inflammatory biomarkers levels between patients with and without chronic immunosuppression. Il: Interleukin; §Aplasia (lymphocytes < 1000/mm3); or corticosteroids (if treatment duration >1 month or if treatment amount >2mg/kg regardless of duration); or HIV (positive serology); AIDS (positive HIV serology and clinical complications: pneumocystis pneumonia, Kaposi’s sarcoma, tuberculosis, toxoplasmosis.
(DOCX)

S3 Table Comparison of AUCs for the prediction of death at day 60. Model *=* Age+ SOFA respi + Lymphocytes, + PCT + Il6/mHLA-DR + % the extent of lung lesion, according to the results Table 3. AUC: Area under the curve, CRP: C-reactive protein, PCT: procalcitonin, Il: Interleukin, mHLA DR: HLA-DR monocytic–expression rate, SOFA: Sequential organ failure assessment.
(DOCX)

## List of abbreviations

AI: artificial intelligence
AUC: Area under the curve
BMI: Body mass index
BNP: brain natriuretic peptid
CCTIRS: Comité Consultatif sur le Traitement de l’Information en matière de Recherche dans le domaine de la Santé
CECIC: comité d’éthique des centres d’investigations clinique
CNIL: Commission nationale de l’informatique et des libertés
COVID-19: Coronavirus Disease 2019 (COVID-19)
CPP: comités de protection des personnes
CRP: C-reactive protein
CT-scan: Computed tomographyv scanner
ICU: intensive care unit
IL: interleukin
IRB: institutional review board
LOS: length of stay
mHLA-DR: human histocompatibility leukocyte antigen expression rate on blood monocytes
OFD: oxygen free days
PCT: Procalcitonin
ROC: receiver operating characteristics
RT-PCR: Reverse transcription polymerase chain reaction
SAPS II: simplified acute physiology score
SARS-CoV2: severe acute respiratory syndrome coronavirus 2
SOFA: sequential organ failure assessment
VAP: ventilator associated pneumonia
VFD: Ventilator free days
