# Supplementary material for: SARS-CoV2 pneumonia patients admitted to the ICU: Analysis according to clinical and biological parameters and the extent of lung parenchymal lesions on chest CT scan, a monocentric observational study
Source: PLoS One. 2024 Sep 19;19(9):e0308014. doi: 10.1371/journal.pone.0308014 (PMC11412649; doi:10.1371/journal.pone.0308014)
Supplement: S1 Table — BMI: Body mass index; SAPS II: Simplified acute physiology score II; SOFA: Sequential organ failure assessment; Il: Interleukin; ICU: Intensive care unit. §Aplasia (lymphocytes < 1000/mm3); or corticosteroids (if treatment duration >1 month or if treatment amount >2mg/kg regardless of duration); or HIV (positive serology); AIDS (positive HIV serology and clinical complications: pneumocystis pneumonia, Kaposi’s sarcoma, tuberculosis, toxoplasmosis. (DOCX) [file pone.0308014.s002.docx]

Table S 1: Characteristics of the whole population before imputation

| **N(%) or median [IQR]** | **All** |
| --- | --- |
| **Number of patients** | 270 |
| **Percentage of lung parenchymal lesions (%)** | 46.9 [30 ; 61] |
| **Time from 1^st^ symptoms to CT-scan (missing=39)** | 8 [5 ; 11] |
| **Time from 1^st^ symptoms to ICU admission, days (missing = 14)** | 9 [7 ; 11.5] |
| **Time from ICU admission to CT scan (missing =29)** | 1[1 ; 3] |
| **Before August 2020 (1st Wave)** | 16 (5.9) |
| **Age, years** | 68.8 [60.5 ; 74.8] |
| **Sex (Male)** | 193 (71.5) |
| **BMI > 30 (kg/m²)** | 119 (44.1) |
| **Comorbidities** |  |
| **Cardiovascular disease** | 53 (19.6) |
| **Chronic respiratory disease** | 20 (7.4) |
| **Chronci renal disease** | 21 (7.8) |
| **Immunosuppression§** | 44 (16.3) |
| **Time from hospital to ICU admission, days** | 2 [1 ; 5] |
| **On admission** |  |
| **SAPS II** | 36 [29 ; 45] |
| **SOFA score** | 5 [3 ; 6] |
| **SOFA score without respiratory item** | 1 [1 ; 3] |
| **SOFA score respiratory item > 2** | 193 (71.5) |
| **SOFA score cardio-vascular > 2** | 34 (12.6) |
| **SOFA score kidney > 2** | 37 (13.7) |
| **PaO2/FiO2 (missing = 6)** | 116 [77.6 ; 165] |
| **Invasive mechanical ventilation** | 31 (11.5) |
| **High-flow nasal cannula** | 168 (62.2) |
| **Vasopressors** | 34 (12.6) |
| **Renal replacement therapy** | 10 (3.7) |
| **Steroids** | 227 (84.1) |
| **Laboratory features** |  |
| **Neutrophils(G/L) (missing = 27)** | 6.8 [4.8 ; 9.8] |
| **Lymphocytes (G/L) (missing = 27)** | 0.7 [0.5 ; 1.1] |
| **Monocytes (G/L) (missing = 27)** | 0.4 [0.2 ; 0.6] |
| **Hematocrit (%) (missing =1)** | 39 [35 ; 42] |
| **Platelets (g/l) (missing =6)** | 245 [196 ; 309.5] |
| **Prothrombin time, % (missing =21)** | 87 [77 ; 93] |
| **D-dimer (ng/mL) (missing =6)** | 1230[776.5 ; 2157.5] |
| **Fibrinogen (g/L) (missing =7)** | 7.2 [6.2 ; 7.9] |
| **Creatininemia (umol/L)** | 84 [67 ; 112] |
| **Bilirubinemia (umol/L) (missing =8)** | 10 [7 ; 13.7] |
| **Lactate (mmol/L) (missing = 3)** | 1.4 [1.1 ; 1.8] |
| **Procalcitonin (ug/L) (missing =17)** | 0.3 [0.1 ; 0.7] |
| **C-reactive protein (mg/L) (missing = 62)** | 113.5 [69.9 ; 168.5] |
| **Ferritin (ug/L) (missing = 22)** | 1164 [680.5 ; 1938] |
| **IL-10 (pg/mL) (missing =15)** | 4.6 [2.3 ; 8.5] |
| **IL-1b (pg/mL) ((missing =17)** | 0 [0 ; 1] |
| **IL-6 (pg/mL) (missing =13)** | 44.6 [16.3 ; 90.6] |
| **mHLA DR (pg/mL) (missing = 45)** | 9180 [6365 ; 13330] |
| **IL-6/mHLA DR x 1000 (missing =48)** | 4.8 [1.6 ; 10.2] |
| **During ICU stay** |  |
| **Vasopressors, n (%)** | 90 (33.3) |
| **Invasive mechanical ventilation, n (%)** | 90 (33.3) |
| **Renal replacement therapy, n (%)** | 46 (17) |
| **Pulmonary embolism, n (%)** | 14 (5.2) |
| **Ventilator-associated pneumonia, n (%)** | 35 (13.1) |
| **Invasive aspergillosis, n (%)** | 13 (4.9) |
| **Ventilatory-free days at day 60** | 60 [0 ; 60] |
| **ICU length of stay, days** | 14.5 [10 ; 24] |
| **Hospital length of stay, days** | 8 [5 ; 13] |
| **Hospital death** | 83 (30.7) |
| **Day-60 mortality** | 92 (34.1) |
