# Supplementary material for: SARS-CoV2 pneumonia patients admitted to the ICU: Analysis according to clinical and biological parameters and the extent of lung parenchymal lesions on chest CT scan, a monocentric observational study
Source: PLoS One. 2024 Sep 19;19(9):e0308014. doi: 10.1371/journal.pone.0308014 (PMC11412649; doi:10.1371/journal.pone.0308014)
Supplement: S2 Table — Il: Interleukin; §Aplasia (lymphocytes < 1000/mm3); or corticosteroids (if treatment duration >1 month or if treatment amount >2mg/kg regardless of duration); or HIV (positive serology); AIDS (positive HIV serology and clinical complications: pneumocystis pneumonia, Kaposi’s sarcoma, tuberculosis, toxoplasmosis. (DOCX) [file pone.0308014.s003.docx]

Table S 2: Comparisons of inflammatory biomarker levels between patients with and without chronic immunosuppression§

|  | Non-immunosuppressed § | Immunosuppressed | P-value |
| --- | --- | --- | --- |
| Number of patients | 226 | 44 |  |
| Extent of lung lesion | 48 [31 ; 62] | 44 [25.5 ; 54.5] | 0.06 |
| Neutrophils (G/L) | 7 [4.9 ; 9.9] | 6.1 [4.1 ; 9.7] | 0.21 |
| Lymphocytes (G/L) | 0.7 [0.5 ; 1.1] | 0.7 [0.4 ; 1.1] | 0.20 |
| Monocytes (G/L) | 0.4 [0.2 ; 0.6] | 0.3 [0.1 ; 0.5] | 0.03 |
| D-dimer (ng/mL) | 1251.5 [786 ; 2246] | 1248.5 [759 ; 2209] | 0.97 |
| Fibrinogen (g/L) | 7.2 [6.2 ; 7.9] | 7.2 [6.6 ; 8.5] | 0.34 |
| Procalcitonin (ug/L) | 0.3 [0.1 ; 0.7] | 0.6 [0.2 ; 2.9] | 0.02 |
| C-reactive protein (mg/L) | 131.5 [83 ; 184] | 125 [72.4 ; 182.5] | 0.69 |
| Ferritin (ug/L) | 1208 [681 ; 2078.6] | 1316 [760.5 ; 2314.5] | 0.51 |
| IL-10 (pg/mL) | 4.6 [2.3 ; 8.3] | 6.6 [3 ; 9.6] | 0.08 |
| IL-1b (pg/mL) | 0 [0 ; 1] | 0.2 [0 ; 2.4] | 0.09 |
| IL-6 (pg/mL) | 44.7 [15.9 ; 95.4] | 56.1 [29.7 ; 125.5] | 0.06 |
| mHLA DR (pg/mL) | 7762.5 [4539 ; 12397] | 7792.5 [3043 ; 11923] | 0.57 |
| IL-6/mHLA DR x 1000 | 5.4 [1.4 ; 12.3] | 6.6 [2.9 ; 20.5] | 0.20 |

Il: Interleukin;

§Aplasia (lymphocytes < 1000/mm3), or corticosteroid treatment (if treatment duration >1 month or if treatment amount >2mg/kg regardless of duration), or HIV (positive serology), and AIDS (positive HIV serology and clinical complications: pneumocystis pneumonia, Kaposi’s sarcoma, tuberculosis, toxoplasmosis
