# Supplementary material for: SARS-CoV2 pneumonia patients admitted to the ICU: Analysis according to clinical and biological parameters and the extent of lung parenchymal lesions on chest CT scan, a monocentric observational study
Source: PLoS One. 2024 Sep 19;19(9):e0308014. doi: 10.1371/journal.pone.0308014 (PMC11412649; doi:10.1371/journal.pone.0308014)
Supplement: S3 Table — Model = Age+ SOFA respi + Lymphocytes, + PCT + Il6/mHLA-DR + % the extent of lung lesion, according to the results Table 3. AUC: Area under the curve, CRP: C-reactive protein, PCT: procalcitonin, Il: Interleukin, mHLA DR: HLA-DR monocytic–expression rate, SOFA: Sequential organ failure assessment. (DOCX) [file pone.0308014.s004.docx]

Table S3: Comparison of AUCs for the prediction of death at day 60.

| PaO2/FiO2 |  |  |  |  |  |  |  |  |
| --- | --- | --- | --- | --- | --- | --- | --- | --- |
| CRP | 0.07 |  |  |  |  |  |  |  |
| PCT | 0.23 | 0.46 |  |  |  |  |  |  |
| Neutrophils | 0.12 | 0.61 | 0.78 |  |  |  |  |  |
| Lymphocytes | 0.02 | 0.71 | 0.28 | 0.43 |  |  |  |  |
| Ddimer | 0.16 | 0.56 | 0.85 | 0.92 | 0.32 |  |  |  |
| HLA-DR / Il6 | 0.33 | 0.26 | 0.78 | 0.56 | 0.17 | 0.67 |  |  |
| % lung lesion | 0.06 | 0.86 | 0.58 | 0.74 | 0.59 | 0.68 | 0.41 |  |
| Model | <0.01 | <0.01 | <0.01 | <0.01 | <0.01 | <0.01 | <0.01 | <0.01 |
| P-value | PaO2/FiO2 | CRP | PCT | Neutrophils | Lymphocytes | Ddimers | HLA-DR . Il6 | % lung lesion |

Model=Age+ SOFA respi + Lymphocytes, + PCT + Il6/mHLA-DR + % the extent of lung lesion, according to the results Table 3.

AUC: Area under the curve, CRP: C-reactive protein, PCT: procalcitonin, Il: Interleukin, mHLA DR: HLA-DR monocytic–expression rate, SOFA: Sequential organ failure assessment.
